# Prolonged grief disorder for ICD-11: the primacy of clinical utility and international applicability

**DOI:** 10.1080/20008198.2018.1476441

**Published:** 2018-06-06

**Authors:** Clare Killikelly, Andreas Maercker

**Affiliations:** Department of Psychology, Division of Psychopathology and Clinical Intervention, University of Zürich, Zürich, Switzerland

**Keywords:** International Classification of Diseases, 11th edition, prolonged grief disorder, clinical utility, international applicability, World Health Organization, Clasificación Internacional de Enfermedades 11a Edición, trastorno por duelo prolongado, utilidad clínica, aplicabilidad internacional, 国际疾病分类第11版, 延长哀伤障碍, 临床实用性, 国际适用性, • We present the new ICD-11 diagnostic criteria for prolonged grief disorder.• We discuss the WHO’s prioritization of clinical utility and how this shaped the new diagnostic criteria.• We review prior and current research evidence supporting the new diagnostic criteria.• We introduce new directions in cross-cultural applicability of the new PGD criteria.

## Abstract

A new mental health disorder, prolonged grief disorder (PGD), will be included in the 11th edition of the International Classification of Diseases (ICD-11). We provide a brief overview of the historical conceptualizations of disordered grief and the previous research efforts to assess and define this condition. We describe the new ICD-11 PGD symptom criteria and how they are conceptualized in terms of the World Health Organization’s call for improved clinical utility. Finally, we review the research evidence for the clinical utility of the new ICD-11 PGD symptom structure and usability in the international arena.

## Abstract (es)

El nuevo trastorno mental, trastorno por duelo prolongado (TDP), será incluido en la decimoprimera edición de la Clasificación Internacional de Enfermedades (CIE-11). Se presenta una breve revisión general de las conceptualizaciones históricas del duelo patológico y los esfuerzos previos en materia de investigación para evaluar y definir esta condición. Se describen los nuevos criterios sintomáticos de CIE-11 para TDP y cómo son conceptualizados en relación al llamado de la Organización Mundial de la Salud (OMS) para favorecer la utilidad clínica. Finalmente, se revisa la evidencia de investigaciones sobre la utilidad clínica de la estructura sintomática del nuevo TDP de la CIE-11 y su aplicabilidad en la esfera internacional.

## Abstract (zh)

国际疾病分类第11版（ICD-11）将包括一个新的心理健康障碍——延长哀伤障碍（PGD）。 我们简要概述了哀伤障碍的历史概念，以及前期评估和定义这种情况的研究工作。 我们描述了新的ICD-11 PGD症状标准，以及如何根据世界卫生组织（WHO）改善临床实用性的呼吁对其进行概念化。 最后，我们回顾了新的ICD-11 PGD症状结构的临床应用，及其在国际范围内使用性的研究证据。

## 1. Introduction

In the proposal for the 11th edition of the International Classification of Diseases (ICD-11), disorders specifically associated with stress have been a topic of great interest for researchers and clinicians, particularly the categories of post-traumatic stress disorder (PTSD) and complex post-traumatic stress disorder (complex PTSD). This paper focuses on the category of prolonged grief disorder (PGD), which is a newcomer to psychopathology and, for the first time, included in a leading classification guideline such as the ICD-11. PGD is characterized by core symptoms such as longing for and preoccupation with the deceased, along with emotional distress and significant functional impairment that persist beyond half a year after the loss of a significant other. Until recently, researchers and clinicians have used different diagnostic criteria and different assessment measures for disordered grief, as the previous diagnostic criteria for PGD had not been established and recognized. This article presents the new ICD-11 diagnostic guideline for PGD. The new guidelines are in line with the requirements and recommendations by the World Health Organization (WHO) for improved clinical utility and international applicability. This debate piece aims to underline the rationale for the new ICD-11 PGD criteria; first, with regard to recommendations by the WHO and, secondly, in terms of previous formative research (Prigerson & Maciejewski, 2017; Reynolds, Cozza, & Shear, 2017).

### 1.1. PGD diagnosis and its precursors

Previous reviews have thoroughly documented the history of PGD (Jordan & Litz, 2014; Maercker & Lalor, 2012; Wagner & Maercker, 2010), and therefore we provide a brief overview relevant to the current ICD-11 PGD criteria. In 1993, Horowitz and colleagues developed the first diagnostic criteria for a bereavement-related disorder, termed ‘pathological’ then ‘complicated’ grief (CG) (Horowitz, Bonanno, & Holen, 1993; Horowitz et al., 1997). It was noted that alongside PTSD, which occurs in response to a traumatic event (or events), patients could experience intrusive preoccupation, avoidance and intense negative emotions after bereavement. Horowitz and colleagues originally conceptualized disordered grief in line with stress-response syndromes; as a reaction to a stressful life event. This ignited research in the field and since this initial introduction and conceptualization, several research groups have used different terminology (pathological, complicated, traumatic, prolonged, chronic or morbid grief), theoretical conceptualizations, assessment measures and diagnostic criteria for a disorder of grief (Wagner & Maercker, 2010). With regard to terminology, in addition to CG, ‘traumatic grief’ was frequently used by Prigerson and colleagues (e.g. Prigerson et al., 1999) as their research uncovered the importance of including core symptoms of separation distress and traumatic distress along with the associated negative emotions. The term PGD was later adopted over ‘complicated’ or ‘traumatic grief’ (along with new diagnostic criteria discussed below) as it was thought to be easier to define and explain, to provide key insight into one important feature of the disorder (the duration of symptoms) and to avoid confusion with PTSD (Wagner & Maercker, 2010).

Disordered grief has also been conceptualized in terms of depression (Clayton, 1990), relational or attachment related (Rubin et al., 2008; Shear et al., 2007) or as subtypes of responses to loss (delayed, unresolved or absent grief) (Stroebe et al., 2000). These various conceptualizations provided valuable insight and strong foundations for the current ICD-11 conceptualization of PGD as distinct from depression with no subtypes and as a stress-related disorder, as discussed in detail in Section 3. With regard to assessment, several questionnaires and measures have been developed for disordered grief (Jordan & Litz, 2014; Maercker & Lalor, 2012). Of note, in 1995, Prigerson and colleagues developed one of the initial assessment questionnaires, the ‘Inventory of Complicated Grief’ (Prigerson et al., 2008, 1995) that became the basis for two sets of diagnostic criteria: PGD-2009 criteria and CG criteria (Horowitz et al., 1997; Prigerson et al., 2009, 1999; Shear et al., 2011). Each will be described below.

In 2009, a broad group of authors developed a consensus on the criteria for a clinical diagnosis of PGD, here termed PGD-2009 criteria (Horowitz et al., 1997; Prigerson et al., 2009, 1999). An analysis of a sample of 317 individuals from the Yale Bereavement Study (Prigerson et al., 2009) generated sensitive and specific items for the PGD-2009 criteria (Table 1). Around the same time as the refinement of the PGD-2009 criteria, Shear and colleagues built on the earlier conceptualization of disordered grief as CG and proposed important mechanisms for the behavioural and biological aetiology (Shear et al., 2007; Zisook & Shear, 2009). Shear and colleagues proposed alternative diagnostic criteria for CG developed from a large clinical sample of treatment-seeking individuals and from a consensus with clinical experts (Reynolds et al., 2017; Shear, 2015; Shear, Jackson, Essock, Donahue, & Felton, 2006; Shear et al., 2011). The PGD-2009 criteria and CG criteria differ in terms of the populations in which they were assessed, the statistical methods used to assess the criteria, the number of items required to receive a diagnosis and descriptions of the items (Maciejewski & Prigerson, 2017; Reynolds et al., 2017). It should be noted that at the time of the revision of the Diagnostic and Statistical Manual of Mental Disorders, 4th Edition (DSM-IV), persistent complex bereavement disorder (PCBD) was introduced as a compromise between the two proposed diagnostic criteria for PGD and CG and placed in section III of the 5th Edition of DSM (DSM-5) as a disorder requiring further study (Maciejewski, Maercker, Boelen, & Prigerson, 2016; Reynolds et al., 2017).

**Table 1.** Comparison of diagnostic criteria for prolonged grief disorder (PGD).

ICD-11 PGD criteria		PGD-2009 criteria	
**A. At least one of the following**	1. Persistent and pervasive longing for the deceased or 2. A persistent and pervasive preoccupation with the deceased	**A. Event**	Bereavement (loss of a significant other)
**B. Examples of intense emotional pain**	Accompanied by intense emotional pain e.g. sadness, guilt, anger, denial, blame Difficulty accepting the death Feeling one has lost a part of one’s self An inability to experience positive mood Emotional numbness Difficulty in engaging with social or other activities	**B. Separation distress**	The bereaved person experiences yearning (e.g. craving, pining or longing for the deceased; physical or emotional suffering as a result of the desired, but unfulfilled, reunion with the deceased) daily or to a disabling degree
**C. Time and impairment criterion**	Persisted for an abnormally long period of time (more than 6 months at a minimum): following the loss, clearly exceeding expected social, cultural or religious norms for the individual’s culture and context. Grief reactions that have persisted for longer periods that are within a normative period of grieving given the person’s cultural and religious context are viewed as normal bereavement responses and are not assigned a diagnosis. The disturbance causes significant impairment in personal, family, social, educational, occupational or other important areas of functioning.	**C. Cognitive, emotional and behavioural symptoms**	The bereaved person must have five (or more) of the following symptoms experienced daily or to a disabling degree: 1. Confusion about one’s role in life or diminished sense of self (i.e. feeling that a part of oneself has died) 2. Difficulty accepting the loss 3. Avoidance of reminders of the reality of the loss 4. Inability to trust others since the loss 5. Bitterness or anger related to the loss 6. Difficulty moving on with life (e.g. making new friends, pursuing interests) 7. Numbness (absence of emotion) since the loss 8. Feeling that life is unfulfilling, empty or meaningless since the loss 9. Feeling stunned, dazed or shocked by the loss
		**D. Timing**	Diagnosis should not be made until at least 6 months have elapsed since the death
		**E. Impairment**	The disturbance causes clinically significant impairment in social, occupational or other important areas of functioning (e.g. domestic responsibilities)
		**F. Relation to other mental disorders**	The disturbance is not better accounted for by major depressive disorder, generalized anxiety disorder or post-traumatic stress disorder

ICD-11, 11th edition of the International Classification of Diseases.

Previous prevalence rates for a disorder of grief vary and are estimated to range from 4.2% in Switzerland, based on the Horowitz et al. (1997) diagnostic criteria for CG (Forstmeier & Maercker, 2007), to 6.7% in a bereaved population sample from Germany, based on the Inventory of Complicated Grief – Revised (ICG-R) (Kersting, Brähler, Glaesmer, & Wagner, 2011), to approximately 9.8% in a 2017 meta-analysis; however, these rates are from individuals who experienced a non-violent/non-traumatic loss (Lundorff, Holmgren, Zachariae, Farver-Vestergaard, & O’Connor, 2017). Conversely, those bereaved by a violent death are likely to have higher rates of a disorder of grief, at approximately 10–15% (Prigerson, 2004) or as high as 14–76% as reported in those who experienced a disaster (Kristensen, Weisæth, & Heir, 2012). It should be noted that these studies provide an approximation of prevalence as they used different instruments and cut-off scores for the assessment measures.

The diagnostic performance of the PGD-2009 criteria has also been well researched. The predictive validity (Prigerson et al., 2009) and the diagnostic distinction of PGD have been consistently confirmed. The symptoms of PGD are found to be distinct from often comorbid near neighbours such as depression, PTSD and separation anxiety (Boelen, van de Schoot, van den Hout, de Keijser, & van den Bout, 2010; Boelen, 2013). Several different statistical methods have been used to confirm the distinct nature of PGD, including confirmatory factor analysis (Boelen, van den Hout, & van den Bout, 2008), latent class analysis (Boelen, Reijntjes, J. Djelantik, & Smid, 2016) and network analysis (Robinaugh, LeBlanc, Vuletich, & McNally, 2014). In light of recent research (Cozza et al., 2016; Mauro et al., 2017), it should be noted that different results have been reported in terms of the diagnostic performance of the PGD-2009 criteria, presumably depending on the study population sample. For example, the original paper (Prigerson et al., 2009) assessed the PGD-2009 in a community sample in the USA, whereas the more recent reports from Cozza et al. (2016) and Mauro et al. (2017) assessed military and clinical samples and found differing rates of diagnostic sensitivity. This attests to the importance of establishing future diagnostic criteria across a variety of samples.

Based on the important research findings outlined above, it is proposed in this debate piece that the precursor criteria, PGD-2009, with the new refinements by the WHO working group, offer valid and clinically useful diagnostic guidelines for the inclusion of PGD in the ICD-11. This is reviewed in detail below.

## 2. New conceptualization of PGD for the ICD-11: the primacy of clinical utility

The main aim of the WHO is to provide the best possible health service and outcomes for individuals worldwide. The topic of grief and loss is of particular importance to the WHO and the ICD-11 as it is used by a large number of countries that are affected by conflict, war and high rates of mortality (Bryant, 2014). The new PGD ICD-11 criteria are conceptualized in line with the key aims of the WHO to improve clinical utility and international applicability. Historically, the revision of diagnostic manuals such as the DSM and ICD has centred on improving the diagnostic specification and reliability of disorder criteria. Although this has improved the identification and treatment of mental disorders, the long and, at times, complicated symptom lists and categories are not always practical in the clinical setting (Hyman, 2007; Keeley et al., 2016; Maj, 2015; Reed, 2010). The framework for the revision of the ICD disorders has fundamentally shifted to prioritizing clinical utility. Clinical utility has three prime components: improved communication, ease of use and treatment planning (First, Reed, Hyman, & Saxena, 2015; Mullins-Sweatt, Lengel, & Deshong, 2016). As outlined by Reed (2010), clinical utility is vital to the effective revision of the ICD, as it will impact the everyday lives of patients and clinicians and the direction of international health. To establish the clinical utility of the proposed new diagnostic criteria for mental and behavioural disorders, the ICD revision group adopted a two-phase research strategy: (1) to develop guidelines for the formative structure and content of a mental disorder; and (2) to evaluate the usability of these guidelines in reaching diagnostic decisions in the international field (Keeley et al., 2016). This was achieved through the use of multiple methods across different studies, for example professional surveys, field studies, case-controlled studies, and ecologically implemented assessments of performance and usability (Keeley et al., 2016). Below, we highlight the relevant results from the above-mentioned research, in support of the ICD-11 PGD criteria (i.e. research supporting the symptom structure of ICD-11 PGD and use in the field) (Table 1).

## 3. Evidence for the clinically useful symptom structure of ICD-11 PGD

The first phase in establishing the clinical utility of a new diagnostic category is the formative phase. The aim of this phase is to establish the guiding principles for the structure and content of a disorder (Keeley et al., 2016). Professional surveys that examine clinicians’ opinions and perspectives on mental disorder classification were conducted alongside field studies to explore clinicians’ use of the disorder criteria and discover areas for improvement. Large surveys of almost 5000 psychiatrists and 2155 psychologists worldwide (Evans et al., 2013; Reed, Mendonça Correia, Esparza, Saxena, & Maj, 2011) explored clinicians’ perspectives on the current and future diagnostic classification systems for all mental and behavioural disorders. The results showed that when considering mental disorders, clinicians have a strong preference for flexible working diagnostic guidelines to allow space for more clinical judgement, more consideration for cultural factors, and a classification system with fewer categories and no subtypes.

The new ICD-11 PGD aligns well with these guiding principles. The structure of the new criteria highlights the following few core symptoms and criteria: yearning and longing, persistent preoccupations, symptoms of intense emotional pain, and significant psychosocial impairment, for a minimum of 6 months, beyond the expected sociocultural norms (Table 1). Conversely, the PGD-2009 criteria (Prigerson et al., 2009) require a minimum of five accessory symptoms in addition to separation distress as a diagnostic threshold. As the new ICD disorder definitions are based on a typological approach, there is no strict requirement for the number of symptoms needed to meet the diagnostic threshold, which will also result in greater sensitivity of case identification. This will allow clinicians more flexibility to use their clinical judgement, which is one of the key recommendations from clinicians (Evans et al., 2013). Another example of increased consideration for clinical judgement is the criterion for ‘significant functional impairment’. This allows for clinical judgement about the nature and extent of impairment, i.e. clinicians may consider cases where functioning is maintained only through significant additional effort. It is evident from Table 1 that the ICD-11 diagnostic criteria and its algorithm are simpler to apply and can better accommodate clinical judgement. The briefer, more straightforward ICD-11 PGD criteria will allow for easier communication with other practitioners, patients and people close to them.

Of note, the call for an increase in cultural considerations is acknowledged in the new ICD-11 criteria, specifically with the reference to the duration, i.e. a long period’ (> 6 months), ‘clearly exceeding expected social, cultural or religious norms for the individual’s culture and context.’ The current research evidence for the importance of this sociocultural caveat is reviewed in Section 4.2, below.

### 3.1. Valid symptom structure

In a 2016 study, the new PGD ICD-11 symptom criteria (of note, this was a shortened item list of two core symptoms and five symptoms of emotional pain) were compared to the PGD-2009 criteria, the PCBD criteria of the DSM-5 and CG through a reanalysis of the Yale Bereavement Study data (Maciejewski et al., 2016). This was a longitudinal interview based study of a sample of 317 bereaved individuals. A high level of agreement between the new ICD-11 criteria, the PGD-2009 criteria and the PCBD criteria was confirmed through psychometric comparison of sensitivity, specificity and predictive validity. However, the structure of CG criteria (Shear et al., 2011) in this particular community-based sample performed poorly in terms of overlap with the other symptom criteria and in terms of predicting health outcomes. A more systematic comparison of PGD (ICD-11) and the alternative CG concept in other (clinical) samples is still pending. Overall, this study confirms that the criteria presented by the ICD-11 provide the same valid symptom structure as the important precursor criteria (e.g. PGD-2009, PCBD); however, these new core criteria are fewer and easier to memorize, and may be more easily applied to diverse clinical contexts around the world.

An independent analysis confirmed the validity of some core components of the PGD-ICD 11 criteria. Network analysis examined the relationship between proposed symptoms of PCBD to isolate and identify the key symptoms central to the disorder (Robinaugh et al., 2014). The most highly correlated symptoms were found to be preoccupation, emotional pain, yearning and feeling that life is empty. These symptoms map directly on to the core criteria of ICD-11 PGD and some of the accessory symptoms, thereby confirming the validity of the new ICD-11 PGD symptom structure. It should be noted that these core components are also similar to the PGD-2009 criteria. Further research is needed to directly compare the PGD-2009 criteria with the ICD-11 criteria using network analysis.

## 4. Evidence for the clinical utility of ICD-11 PGD in the field

According to the WHO’s research strategy for clinical utility, after the first phase (the formative phase to establish the guiding principles for the structure and content of a disorder), the second phase is to evaluate the use of the criteria in the field. This is to ensure that clinicians are able to apply the new criteria in a usable and valid form. This was done through case-controlled studies that used experimental methods comparing standardized vignettes with different diagnostic criteria and through the assessment of the proposed diagnostic criteria in the clinic with patients (Keeley et al., 2016).

### 4.1. Case identification

One of the first indications from the field of the clinical utility of the ICD-11 PGD was shown in the international ecological implementation field study organized by the WHO by means of the Global Clinical Practitioners Network (https://gcp.network/en/) (Keeley et al., 2016). In this study, 1738 clinicians representing 76 different countries were presented with a series of standardized case vignettes to test important differences in the proposed diagnostic criteria for ICD-11 compared with ICD-10. With regard to PGD, clinicians assessed whether ‘PGD could be differentiated from a normal grief response based on the proposed ICD-11 diagnostic guidelines’. The field test confirmed that under the ICD-11 criteria clinicians could accurately and reliably diagnosis PGD in the appropriate vignette with 92% agreement, which is regarded as particularly satisfactory. Thus, from the first field tests the proposal for the ICD-11 guidelines performed well.

### 4.2. International applicability

In the development of the ICD-11 diagnostic guidelines, special attention is given to the inclusion of culture-related features; for example, the clinical description of PGD in the ICD-11 includes caveats about cultural differences in the duration of symptoms and the expression of grief. In fact, a diagnosis of PGD cannot be ascertained unless it is clear that the symptoms violate current sociocultural norms.

In the current ICD-11 clinical description of PGD, the duration of the grief response is not limited to a specific 6 month time frame. This caveat, in part, originates from WHO’s above-mentioned vignette implementation field study (Keeley et al., 2016). In this field study, some clinicians diagnosed PGD in a vignette that was meant to represent a normal bereavement response at less than 6 months post-loss. There was disagreement over the duration of the grief response and whether or not it was culturally appropriate.

In general, the ICD-11 intends to give duration numbers (such as 6 months) to provide clinicians with an approximation, in an effort not to discount the broad variability of human reactions (Reed, 2010). As yet, the best time-point for distinguishing PGD from normal bereavement has not been scientifically validated. However, strong indications suggest that beyond 6 months is an appropriate time frame (Prigerson et al., 2009; Shear et al., 2011). The ICD-11 PGD definition considers that the duration of the grieving process varies considerably in different cultural and social settings. For example, in Taiwan, widows are highly discouraged from crying in front of the recently deceased (Rosenblatt, 2008), and in societies with a dominant Christian tradition, a period of mourning lasting for one year – e.g. in Germany the ‘mourning year’ (*Trauerjahr*) – is generally culturally acceptable (for a review see Hays & Hendrix, 2008). This mourning period extends beyond the ICD-11 PGD time frame of 6 months and supports the inclusion of a cultural caveat, in this case, within a society of the ‘global north’. Religion, social status, gender and other features of mourning distinctively influence the duration of the expression of grief. The new ICD-11 guidelines include specific reference to violation of sociocultural norms as a clear step towards increased international applicability. It should be noted that the DSM-5 also includes cultural caveats in the form of the cultural formulation interview, and in the additional criteria section of the PCBD diagnostic criteria there is reference to inconsistency with cultural or religious norms.

Along with cultural differences in the duration of the grief response, there may also be differences in the symptom content of normal or pathological grief. Hinton, Field, Nickerson, Bryant, and Simon (2013) found that for Cambodian refugees the need to re-experience the dead through dreams or visions was a crucial part of normal bereavement, whereas in Western culture visions and hallucinations may be considered pathological. The inclusion of cultural caveats in the new ICD-11 criteria provides clinicians with flexibility and encourages cultural sensitivity when considering diagnosis.

Indirect and direct evidence for the international application of the ICD-11 PGD criteria comes from a steadily increasing body of research on cross-cultural understanding of grief. Since the initial proposal of the ICD-11 criteria (Maercker et al., 2013), 17 studies have adapted different measures of grief (e.g. PG-13, ICD-11 criteria, ICG-R) for use in at least 10 different countries, including India, Iraq and East Timor. Xiu et al. (2016) compared PGD symptom profiles using the PG-13 plus some additional items to align with the ICD-11 criteria, in China and Switzerland, and found that grief symptoms were predominantly similar. However, bereaved people in Switzerland presented with somewhat higher grief-related preoccupation, a core symptom, while the bereaved Chinese participants presented with greater functional impairment. This indicates that the ICD-11 criteria can be used cross-culturally to note similarities and differences in the experience of grief.

### 4.3. Therapeutic and interventional implications

The ultimate goal of diagnostic assessment is to identify those in need of support and to provide advice on the best available treatment. PGD can be regarded as a common mental health diagnosis that previously may have appeared under different labels, such as depressive or somatoform (bodily distress) disorders (Maercker, Neimeyer, & Simiola, 2016). It is foreseeable that global recognition of the diagnosis of PGD will improve the detection and prevalence of PGD and increase the need to treat this condition in a specific way. The WHO Mental Health Gap (mhGAP) programme includes bereavement as a key target for the development of worldwide interventions and treatment (WHO, 2016). Its guidelines can be used for assessment and treatment, particularly the recommendations that the management of grief symptoms should include the discussion of culturally appropriate adjustment and mourning rituals. In addition, there have been several successful randomized controlled trials of treatments for CG (Shear, Frank, Houck, & Reynolds, 2005; Shear et al., 2016, 2014) and PGD (Bryant et al., 2014; Kersting et al., 2013; Litz et al., 2014; Rosner, Pfoh, Kotoučová, & Hagl, 2014). These treatments pull valuable techniques from PTSD interventions, narrative therapy and cognitive behavioural therapy, and could be incorporated into the WHO mhGAP guidelines (Rosner, Bartl, Pfoh, Kotoučová, & Hagl, 2015; Shear, 2015).

The success of the ICD-11 criteria and the WHO guidelines depends on how well these translate to clinical practice. A programmatic paper highlighted how there may be challenges in implementing the new ICD-11 guidelines, particularly in low- and middle-income countries (Tol et al., 2014). First, there has been limited research worldwide into effective interventions for disorders specifically associated with stress – to which PGD belongs. There is insufficient research evidence for or against current interventions for acute stress that may be similar to PGD interventions (psychoeducation, relaxation, problem-solving counselling). This is particularly true for children and adolescents, and for individuals from countries in the global south. When considering low- and middle-income countries, where there is a high prevalence of stress-related disorders, these challenges are compounded by a lack of resources and a potential reluctance to adopt new practices without increased support (Tol et al., 2014). The development and strengthening of an evidence base for effective treatment of stress-related disorders, including PGD, should be the next important step, in which partnerships with humanitarian organizations, research institutions and non-governmental organizations will be of particular importance.

## 5. Future directions

As PGD is a newly refined diagnostic category, ultimately, clinic-based field tests and epidemiological studies are required to confirm its clinical utility. There are still significant questions concerning the application of this definition to different cultural groups and across the lifespan (Keeley et al., 2016; Reed et al., 2011; Robles et al., 2014; Schaal, Jacob, Dusingizemungu, & Elbert, 2010). To date, the WHO framework of clinical utility and international applicability has contributed to the development of applicable and culturally informed diagnostic criteria for PGD that, it is hoped, will improve clinical practice.

## References

[CIT0001] Boelen P. A. (2013). Symptoms of prolonged grief, depression, and adult separation anxiety: Distinctiveness and correlates. *Psychiatry Research*, 207(1–2), 68–9. 23068081 10.1016/j.psychres.2012.09.021

[CIT0002] Boelen P. A., Reijntjes A., J. Djelantik A. A. A. M., & Smid G. E. (2016). Prolonged grief and depression after unnatural loss: Latent class analyses and cognitive correlates. *Psychiatry Research*, 240, 358–363. 27138832 10.1016/j.psychres.2016.04.012

[CIT0003] Boelen P. A., van de Schoot R., van den Hout M. A., De Keijser J., & van den Bout J. (2010). Prolonged Grief Disorder, depression, and posttraumatic stress disorder are distinguishable syndromes. *Journal of Affective Disorders*, 125(1–3), 374–378. 20189657 10.1016/j.jad.2010.01.076

[CIT0004] Boelen P. A., van den Hout M. A., & van den Bout J. (2008). The factor structure of Posttraumatic Stress Disorder symptoms among bereaved individuals: A confirmatory factor analysis study. *Journal of Anxiety Disorders*, 22(8), 1377–1383. 18342486 10.1016/j.janxdis.2008.01.018

[CIT0005] Bryant R. A. (2014). Prolonged grief. *Current Opinion in Psychiatry*, 27(1), 21–26. 24270486 10.1097/YCO.0000000000000031

[CIT0006] Bryant R. A., Kenny L., Joscelyne A., Rawson N., Maccallum F., Cahill C., … Nickerson A. (2014). Treating prolonged grief disorder: A randomized clinical trial. *JAMA Psychiatry*, 71(12), 1332–1339. 25338187 10.1001/jamapsychiatry.2014.1600

[CIT0007] Clayton P. J. (1990). Bereavement and depression. *Journal of Clinical Psychiatry*, 51(7), 34–38. 2195011

[CIT0008] Cozza S. J., Fisher J. E., Mauro C., Zhou J., Ortiz C. D., Skritskaya N., … Shear K. (2016). Performance of DSM-5 persistent complex bereavement disorder criteria in a community sample of bereaved military family members. *American Journal of Psychiatry*, 173(9), 919–929. 27216262 10.1176/appi.ajp.2016.15111442

[CIT0009] Evans S. C., Reed G. M., Roberts M. C., Esparza P., Watts A. D., Correia J. M., … Saxena S. (2013). Psychologists’ perspectives on the diagnostic classification of mental disorders: Results from the WHO-IUPsyS Global Survey. *International Journal of Psychology*, 48(3), 177–193. 23750927 10.1080/00207594.2013.804189 PMC3725658

[CIT0010] First M. B., Reed G. M., Hyman S. E., & Saxena S. (2015). The development of the ICD-11 clinical descriptions and diagnostic guidelines for mental and behavioural disorders. *World Psychiatry: Official Journal of the World Psychiatric Association (WPA)*, 14(1), 82–90. 25655162 10.1002/wps.20189 PMC4329901

[CIT0011] Forstmeier S., & Maercker A. (2007). Comparison of two diagnostic systems for Complicated Grief. *Journal of Affective Disorders*, 99(1), 203–211. 17055064 10.1016/j.jad.2006.09.013

[CIT0012] Hays J., & Hendrix C. (2008). The role of religion in bereavement In Stroebe M., Hansson R., Schut H., & Stroebe W. (Eds.), *Handbook of bereavement research and practice: Advances in theory and intervention* (pp. 327–348). Washington, DC: American Psychological Association.

[CIT0013] Hinton D. E., Field N. P., Nickerson A., Bryant R. A., & Simon N. (2013). Dreams of the dead among Cambodian Refugees: Frequency, phenomenology, and relationship to complicated grief and posttraumatic stress disorder. *Death Studies*, 37(8), 750–767. 24521031 10.1080/07481187.2012.692457

[CIT0014] Horowitz M., Bonanno G., & Holen A. (1993). Pathological grief: Diagnosis and explanation. *Psychosomatic Medicine*, 55(3), 260–273. 8346334 10.1097/00006842-199305000-00004

[CIT0015] Horowitz M., Siegel B., Holen A., Bonanno G., Milbrath C., & Stinson C. (1997). Diagnostic criteria for complicated grief disorder. *American Journal of Psychiatry*, 154(7), 904–910. 9210739 10.1176/ajp.154.7.904

[CIT0016] Hyman S. E. (2007). Can neuroscience be integrated into the DSM-V? *Nature Reviews Neuroscience*, 8(9), 725–732. 17704814 10.1038/nrn2218

[CIT0017] Jordan A. H., & Litz B. T. (2014). Prolonged grief disorder: Diagnostic, assessment, and treatment considerations. *Professional Psychology: Research and Practice*, 45(3), 180–187.

[CIT0018] Keeley J. W., Reed G. M., Roberts M. C., Evans S. C., Medina-Mora M. E., Robles R., … Saxena S. (2016). Developing a science of clinical utility in diagnostic classification systems: Field study strategies for ICD-11 mental and behavioral disorders. *American Psychologist*, 71(1), 3–16. 26766762 10.1037/a0039972

[CIT0019] Keeley J. W., Reed G. M., Roberts M. C., Evans S. C., Robles R., Matsumoto C., … Maercker A. (2016). Disorders specifically associated with stress: A case-controlled field study for ICD-11 mental and behavioural disorders. *International Journal of Clinical and Health Psychology*, 16(2), 109–127. 10.1016/j.ijchp.2015.09.002 PMC6225017 30487855

[CIT0020] Kersting A., Brähler E., Glaesmer H., & Wagner B. (2011). Prevalence of complicated grief in a representative population-based sample. *Journal of Affective Disorders*, 131(1–3), 339–343. 21216470 10.1016/j.jad.2010.11.032

[CIT0021] Kersting A., Dölemeyer R., Steinig J., Walter F., Kroker K., Baust K., & Wagner B. (2013). Brief internet-based intervention reduces posttraumatic stress and prolonged grief in parents after the loss of a child during pregnancy: A randomized controlled trial. *Psychotherapy and Psychosomatics*, 82(6), 372–381. 24061387 10.1159/000348713

[CIT0022] Kristensen P., Weisæth L., & Heir T. (2012). Bereavement and mental health after sudden and violent losses: A review. *Psychiatry*, 75(1), 76–97. 22397543 10.1521/psyc.2012.75.1.76

[CIT0023] Litz B. T., Schorr Y., Delaney E., Au T., Papa A., Fox A. B., … Prigerson H. (2014). A randomized controlled trial of an internet-based therapist-assisted indicated preventive intervention for prolonged grief disorder. *Behaviour Research and Therapy*, 61, 23–34. 25113524 10.1016/j.brat.2014.07.005 PMC4172488

[CIT0024] Lundorff M., Holmgren H., Zachariae R., Farver-Vestergaard I., & O’Connor M. (2017). Prevalence of prolonged grief disorder in adult bereavement: A systematic review and meta-analysis. *Journal of Affective Disorders*, 212, 138–149. 28167398 10.1016/j.jad.2017.01.030

[CIT0025] Maciejewski P., Maercker A., Boelen P. A., & Prigerson H. (2016). “Prolonged grief disorder” and “persistent complex bereavement disorder”, but not “complicated grief”, are one and the same diagnostic entity: An analysis of data from the Yale Bereavement Study. *World Psychiatry*, 15(3), 266–275. 27717273 10.1002/wps.20348 PMC5032512

[CIT0026] Maciejewski P., & Prigerson H. (2017). Prolonged, but not complicated, grief is a mental disorder. *The British Journal of Psychiatry*, 211(4), 189–191. 28970298 10.1192/bjp.bp.116.196238

[CIT0027] Maercker A., Brewin C. R., Bryant R. A., Cloitre M., Reed G. M., van Ommeren M., … Saxena S. (2013). Proposals for mental disorders specifically associated with stress in the International Classification of Diseases-11. *The Lancet*, 381(9878), 1683–1685. 10.1016/S0140-6736(12)62191-6 23583019

[CIT0028] Maercker A., & Lalor J. (2012). Diagnostic and clinical considerations in prolonged grief disorder. *Dialogues in Clinical Neuroscience*, 14(2), 167–176. 22754289 10.31887/DCNS.2012.14.2/amaercker PMC3384445

[CIT0029] Maercker A., Neimeyer R. A., & Simiola V. (2016). Depression and complicated grief In J. Cook, S. Gold, & C. Dalenberg (Eds.), *APA handbook of trauma psychology.* Washington, DC: American Psychological Association.

[CIT0030] Maj M. (2015). The media campaign on the DSM-5: Recurring comments and lessons for the future of diagnosis in psychiatric practice. *Epidemiology and Psychiatric Sciences*, 24(3), 197–202. 25204198 10.1017/S2045796014000572 PMC6998454

[CIT0031] Mauro C., Shear M. K., Reynolds C. F., Simon N. M., Zisook S., Skritskaya N., … Glickman K. (2017). Performance characteristics and clinical utility of diagnostic criteria proposals in bereaved treatment-seeking patients. *Psychological Medicine*, 47(4), 608–615. 27821201 10.1017/S0033291716002749

[CIT0032] Mullins-Sweatt S. N., Lengel G. J., & Deshong H. L. (2016). The importance of considering clinical utility in the construction of a diagnostic manual. *Annual Reviews Clinical Psychologist*, 12, 133–155. 10.1146/annurev-clinpsy-021815-092954 26666967

[CIT0033] Prigerson H. (2004). Complicated grief. *Bereavement Care*, 23(3), 38–40.

[CIT0034] Prigerson H., Horowitz M. J., Jacobs S. C., Parkes C. M., Aslan M., Goodkin K., … Maciejewski P. K. (2009). Prolonged grief disorder: Psychometric validation of criteria proposed for DSM-V and ICD-11. *PLoS Medicine*, 6(8), e1000121. 19652695 10.1371/journal.pmed.1000121 PMC2711304

[CIT0035] Prigerson H., & Maciejewski P. (2017). Rebuilding consensus on valid criteria for disordered grief. *JAMA Psychiatry*, 74(5), 435–436. 28355449 10.1001/jamapsychiatry.2017.0293 PMC5499154

[CIT0036] Prigerson H., Maciejewski P. K., Reynolds C. F., Bierhals A. J., Newsom J. T., Fasiczka A., … Miller M. (1995). Inventory of complicated grief: A scale to measure maladaptive symptoms of loss. *Psychiatry Research*, 59(1–2), 65–79. 8771222 10.1016/0165-1781(95)02757-2

[CIT0037] Prigerson H., Shear M. K., Jacobs S. C., Reynolds C. F., Maciejewski P. K., Davidson J. R. T., … Zisook S. (1999). Consensus criteria for traumatic grief: A preliminary empirical test. *British Journal of Psychiatry*, 174(JAN), 67–73. 10211154 10.1192/bjp.174.1.67

[CIT0038] Prigerson H., Vanderwerker L. C., & Maciejewski P. K. (2008). A case for inclusion of prolonged grief disorder in DSM-V. *Handbook of Bereavement Research and Practice: Advances in Theory and Intervention*, 165–186. doi:10.1037/14498-008

[CIT0039] Reed G. M. (2010). Toward ICD-11: Improving the clinical utility of WHO’s International Classification of mental disorders. *Professional Psychology: Research and Practice*, 41(6), 457–464.

[CIT0040] Reed G. M., Mendonça Correia J., Esparza P., Saxena S., & Maj M. (2011). The WPA-WHO global survey of psychiatrists’ attitudes towards mental disorders classification. *World Psychiatry: Official Journal of the World Psychiatric Association (WPA)*, 10(2), 118–131. 21633689 10.1002/j.2051-5545.2011.tb00034.x PMC3105474

[CIT0041] Reynolds C. F., Cozza S. J., & Shear K. (2017). Clinically relevant diagnostic criteria for a persistent impairing grief disorder putting patients first. *JAMA Psychiatry*, 74(5), 433–437. 28355457 10.1001/jamapsychiatry.2017.0290

[CIT0042] Robinaugh D. J., LeBlanc N. J., Vuletich H. A., & McNally R. J. (2014). Network analysis of persistent complex bereavement disorder in conjugally bereaved adults. *Journal of Abnormal Psychology*, 123(3), 510–522. 24933281 10.1037/abn0000002 PMC4170793

[CIT0043] Robles R., Fresán A., Evans S. C., Lovell A. M., Medina-Mora M. E., Maj M., & Reed G. M. (2014). Problematic, absent and stigmatizing diagnoses in current mental disorders classifications: Results from the WHO-WPA and WHO-IUPsyS Global Surveys. *International Journal of Clinical and Health Psychology*, 14(3), 165–177.

[CIT0044] Rosenblatt P. C. (2008). Grief across cultures: A review and research agenda In Stroebe M., Hansson R., Schut H., & Stroebe W. (Eds.), *Handbook of bereavement research and practice: Advances in theory and intervention* (pp. 207–222). Washington, DC: American Psychological Association. doi:10.1037/14498-010

[CIT0045] Rosner R., Bartl H., Pfoh G., Kotoučová M., & Hagl M. (2015). Efficacy of an integrative CBT for prolonged grief disorder: A long-term follow-up. *Journal of Affective Disorders*, 183, 106–112. 26001670 10.1016/j.jad.2015.04.051

[CIT0046] Rosner R., Pfoh G., Kotoučová M., & Hagl M. (2014). Efficacy of an outpatient treatment for prolonged grief disorder: A randomized controlled clinical trial. *Journal of Affective Disorders*, 167, 56–63. 25082115 10.1016/j.jad.2014.05.035

[CIT0047] Rubin, S. S., Malkinson, R., Witztum, E., Stroebe, M. S., Hansson, R. O., Schut, H., & Stroebe, W (2008). Clinical aspects of a DSM complicated grief diagnosis: Challenges, dilemmas, and opportunities. In M. Stroebe, R. Hansson, H. Schut, & W. Stroebe (Eds.), *Handbook of bereavement research and practice: Advances in theory and intervention* (pp. 187–206). Washington: American Psychological Association.

[CIT0048] Schaal S., Jacob N., Dusingizemungu J. P., & Elbert T. (2010). Rates and risks for prolonged grief disorder in a sample of orphaned and widowed genocide survivors. *BMC Psychiatry*, 10(1), 55. 20604936 10.1186/1471-244X-10-55 PMC2914709

[CIT0049] Shear K. (2015). Complicated Grief. *New England Journal of Medicine*, 372(2), 153–160. 25564898 10.1056/NEJMcp1315618

[CIT0050] Shear K., Frank E., Houck P. R., & Reynolds C. F. (2005). Treatment of Complicated Grief. *JAMA*, 293(21), 2601. 15928281 10.1001/jama.293.21.2601 PMC5953417

[CIT0051] Shear K., Jackson C. T., Essock S. M., Donahue S. A., & Felton C. J. (2006). Screening for complicated grief among Project Liberty service recipients 18 months after September 11, 2001. *Psychiatric Services*, 57(9), 1291–1297. 16968758 10.1176/ps.2006.57.9.1291

[CIT0052] Shear K., Monk T., Houck P., Melhem N., Frank E., Reynolds C., & Sillowash R. (2007). An attachment-based model of complicated grief including the role of avoidance. *European Archives of Psychiatry and Clinical Neuroscience*, 257(8), 453–461. 17629727 10.1007/s00406-007-0745-z PMC2806638

[CIT0053] Shear K., Reynolds C. F., Simon N. M., Zisook S., Wang Y., Mauro C., … Skritskaya N. (2016). Optimizing treatment of complicated grief: A randomized clinical trial. *JAMA Psychiatry*, 73(7), 685–694. 27276373 10.1001/jamapsychiatry.2016.0892 PMC5735848

[CIT0054] Shear K., Simon N., Wall M., Zisook S., Neimeyer R., Duan N., … Keshaviah A. (2011). Complicated grief and related bereavement issues for DSM-5. *Depression and Anxiety*, 28(2), 103–117. 21284063 10.1002/da.20780 PMC3075805

[CIT0055] Shear K., Wang Y., Skritskaya N., Duan N., Mauro C., & Ghesquiere A. (2014). Treatment of complicated grief in elderly persons: A randomized clinical trial. *JAMA Psychiatry*, 71(11), 1287–1295. 25250737 10.1001/jamapsychiatry.2014.1242 PMC5705174

[CIT0056] Stroebe M., Van Son M., Stroebe W., Kleber R., Schut H., & van den Bout J. (2000). On the classification and diagnosis of pathological grief. *Clinical Psychology Review*, 20(1), 57–75. 10660828 10.1016/s0272-7358(98)00089-0

[CIT0057] Tol W. A., Barbui C., Bisson J., Cohen J., Hijazi Z., Jones L., … Smith K. (2014). World Health Organization guidelines for management of acute stress, PTSD, and Bereavement: Key challenges on the road ahead. *PLoS Medicine*, 11(12), e1001769. 25514024 10.1371/journal.pmed.1001769 PMC4267806

[CIT0058] Wagner B., & Maercker A. (2010). The diagnosis of complicated Grief as a mental disorder: A critical appraisal. *Psychologica Belgica*, 50(1–2), 27.

[CIT0059] WHO (2016). *MhGAP Intervention Guide for mental, neurological and substance use disorders in non-specialized health settings: Mental Health Gap Action Programme (mhGAP): Version 2.0*. Geneva: World Health Organization. 27786430

[CIT0060] Xiu D., Maercker A., Woynar S., Geirhofer B., Yang Y., & Jia X. (2016). Features of prolonged Grief symptoms in Chinese and Swiss bereaved parents. *The Journal of Nervous and Mental Disease*, 204(9), 693–701. 27253073 10.1097/NMD.0000000000000539

[CIT0061] Zisook S., & Shear K. (2009). Grief and bereavement: What psychiatrists need to know. *World Psychiatry: Official Journal of the World Psychiatric Association (WPA)*, 8(2), 67–74. 19516922 10.1002/j.2051-5545.2009.tb00217.x PMC2691160

